# CRISPER/CAS System, a Novel Tool of Targeted Therapy of Drug-Resistant Lung Cancer

**DOI:** 10.34172/apb.2022.027

**Published:** 2021-04-03

**Authors:** Vahid Akbari Kordkheyli, Mohsen Rashidi, Yasaman Shokri, Samane Fallahpour, Atena Variji, Ehsan Nabipour Ghara, Sayed Mostafa Hosseini

**Affiliations:** ^1^Student Research Committee, Faculty of Medicine, Mazandaran University of Medical Sciences, Sari, Iran.; ^2^Department of Pharmacology, Faculty of Medicine, Mazandaran University of Medical Sciences, Sari, Iran.; ^3^Department of Clinical Biochemistry and Genetics, Faculty of Medicine, Mazandaran University of Medical Sciences, Sari, Iran.; ^4^Department of Biochemistry-Genetic and Plant Biology, Faculty of Basic Sciences, Islamic Azad University, Damghan Branch, Damghan, Iran.; ^5^Human Genetic Research Center, Baqiyatallah University of Medical Sciences, Tehran, Iran.

**Keywords:** CRISPR/CAS, Lung, Cancer, Genetic modification

## Abstract

Lung cancer (LC) is the most common cause of cancer-related death worldwide. Patients with LC are usually diagnosed at advanced phases. Five-year survival rate in LC patients is approximately 16%. Despite decades of research on LC treatments, clinical outcomes are still very poor, necessitating to develop novel technologies to manage the disease. Considering the role of genetic and epigenetic changes in oncogenes and tumor-suppressor genes in cancer progression, gene therapy provides a hot spot in cancer treatment research. Gene therapy offers less side effects compared to conventional methods such as chemotherapy. Unlike the traditional approaches of gene therapy that have temporary effects, using genetic modification tools can offer persistent cure. Over the past a few years, many studies have effectively used the CRISPR–Cas9 approach to modify gene expression in cells. This system is applied to induce site-specific mutagenesis and epigenetic modifications and regulate gene expression. In this review, we discuss recent applications of the CRISPR–Cas9 technology in treating LC.

## Introduction

Lung cancer (LC) is the most common cause of cancer-related death in both women and men worldwide. About 1.8 million cases (12.9% of all cancers) and 1.59 million deaths (19.4% of all cancer-related deaths) are linked to LC. Some risk factors of LC include tobacco smoking, air pollution, and exposure to environmental hazards and toxins such as radon, asbestos, arsenic, chromium, nickel, and radiation. Furthermore, underlying lung disorders such as chronic obstructive pulmonary disease, emphysema, chronic bronchitis, pneumonia, and tuberculosis have been associated with the increased risk of LC. This cancer is usually (85% of cases) diagnosed at advanced phases. The rate of five-year survival in patients with advanced LC is approximately 16% while this reaches above 50% in those who are diagnosed at early phases. According to histological features, LC is categorized into non-small-cell lung cancer (NSCLC) and small-cell lung cancer (SCLC). NSCLC is the most common type (80–90%) of all lung cancers and is divided into squamous cell carcinoma (SCC) (or epidermoid carcinoma), adenocarcinoma (bronchioloalveolar), and large cell carcinoma. Adenocarcinoma is the most prevalent (70-80%) type of NSCLC followed by large cell carcinoma. SCC is infrequent among never-smokers; however, bronchial carcinoid, another neuroendocrine tumor, is more prevalent in the never-smokers diagnosed with LC.^1-4^

Despite decades of research on LC treatment, clinical outcomes remain very poor, demanding novel therapeutic technologies to manage the disease. Recently, identification of several genetic mutations in EGFR, KRAS, and ALK in LC has helped to design more specific and targeted treatments. Gene therapy is now considered as the hot spot of cancer treatment research particularly because of having less side effects compared to conventional methods such as chemotherapy. Unlike traditional approaches of gene therapy, novel methods by manipulating the genes involved in tumorigenesis have shown promising and durable results in terms of eradicating tumors.^5-8^

Zinc-finger nucleases (ZFNs), transcription activator-like effector nucleases (TALENs), and the clustered regularly interspaced short palindromic repeats (CRISPR) system are the most common genome engineering technologies which employ site-specific endonucleases. The utilization of ZFNs and TALENs has been restricted due to their costs and the complexity of designing endonucleases. On the other hand, an important advantage of the CRISPR system is that this system targets genomic sequences by a single-guide RNA (sgRNA) instead of protein-based detection used in ZFNs and TALENs (Table 1). Also, CRISPR can simultaneously target different genes employing different sgRNAs. The advent of streptococcus pyogenes-derived CRISPR–Cas (a CRISPR-associated system) in mammalian cells has dramatically changed the perspective towards genome editing approaches. This system consists of Cas9 (RNA-guided DNA endonuclease) and a sgRNA that itself includes a CRISPR-RNA (crRNA) and a trans-activating crRNA (trac-rRNA), joining to and linking the Cas9 to target genes. Many studies in the past few years have shown the efficiency of the CRISPR–Cas9 system in modifying genes in different cells. This system has been applied to modify site-specific mutagenesis, gene expression, and epigenetic signatures, as well as to target RNAs and specific DNA sequences for diagnostic purposes.^9-11^

In this review, we have discussed the recent applications of the CRISPR–Cas9 technology in treating LC.

**Table 1:** The specifications of ZFN, TALENs, and CRISPR genome editing techniques

	**ZFN**	**TALENs**	**CRISPR**
Site-specific endonuclease enzymes	FokI	FokI	Cas9
Type of DNAidentification	protein-DNA interaction	protein-DNA interaction	RNA-DNA interaction
DNA binding factor	triplet-confined zinc finger proteins	single-base recognition TALE proteins	sgRNA
Target region	18–36 bp; guanine rich loci	30–40 bp; 5′ targeted base must be a T	22 bp; PAM sequence
Efficiency/success rate	Low	Moderate	High
Multiplexing capability	No	No	Yes
Cytotoxicity effects	High	Moderate	Low
Engineering complexity	High	Moderate	Easy
Cost	Expensive	Cheaper than ZFN	Cheapest

ZFN: Zinc-finger nucleases, TALEN: transcription activator-like effector nucleases, CRISPR: clustered regularly interspaced short palindromic repeats, sgRNA: single-guide RNA

## Genetics changes in LC

Many genetic mutations increase the risk of cancer. While point and isolated mutations generally exert slight effects, combinations of several mutations can remarkably boost the risk of diseases such as cancer. In LC, multiple mutations in oncogenes and tumor suppressor genes have been observed (Table 2). Among the most observed genetic alterations in LC are the deletion of the short arm of chromosome 3 and duplications of chromosomes 1 and 12. The loss of alleles on chromosome 3 has been detected in about 90% of SCLC and 50% of NSCLC cases.^12-14^ The main oncogenes activated in NSCLC are epidermal growth factor receptor, anaplastic lymphoma kinase (ALK), Myc, Bcl-2, and KRAS. Common altered tumor-suppressor genes include TP53, p16, and retinoblastoma (RB1). These mutations are associated with a poor prognosis in patients. EGFR and ALK, as the members of the tyrosine kinase receptors superfamily, contribute to various cellular processes such as proliferation and differentiation. Mutations in these receptors can lead to ligand-independent activity and subsequently to malignancy. Among the genes mutated in SCLC, TP53 and RB1 tumor suppressors are nearly universal. Furthermore, the methylation/inactivation of RASSF1A tumor suppressor at 3p21 has been reported in 70-100% of SCLCs and 45-65% of NSCLCs. Other genes that may be deleted at 3p region include RARB, caveolin-1, FHIT, and β-catenin. Additional genetic alterations may occur in histone acetyltransferases including CREBBP and EP300, PTEN tumor suppressor, NOTCH family genes, and MYC-related genes.^1,5,12,15^

**Table 2:** Common genetic changes in lung cancer

**Gene aberration**	**SCLC (%)**	**LADC (%)**	**SCC (%)**
Oncogene activation			
EGFR	Rare	10-75	2-9
KRAS	Rare	4-35	1-5
BRAF	Rare	1-5	0.2-2
MET	1.6-13	1-15	5-24
HER2	Rare	1-10	0-1
Tumor-suppressor alterations			
LKB1	Rare	8-50	1-30
TP53	73-90	31-70	46-73
PTEN	5-20	0-5	2.5-11
RB	60-90	0-8	0-16
P16	6	24-60	47-78
Chromosomal rearrangement			
ALK-EML4 fusion	Rare	2.4-16.3	0-2.5
RET fusion	Rare	0.4-2	Rare
ROS1 fusion	Rare	0.9-3	0-1
NRG1 fusion	Rare	0.5-1.7	Rare

It has been demonstrated that 3p107 (RBl1) and 3p130 (RBl2) deletions which are observed in most human SCLCs are particularly associated with tumor progression. The downregulation of SIRT6 has been associated with hyperproliferation in NSCLC. SIRT6 suppresses Twist1 expression, which is a transcription factor involved in cellular differentiation and development. It has also been noted that variations in telomerase reverse transcriptase gene increase susceptibility to all types of LC.^12,16^ Furthermore, mutations in the cyclin-dependent kinase inhibitor 2A (CDKN2) gene (located at 9p21) may lead to SCC while TP63 variants have been implicated in susceptibility to lung adenocarcinoma, especially among East Asian populations.^17,18^ While KRAS mutations are common among smoker LC patients, ALK translocations and EGFR mutations are more frequent among never-smoker patients.^17,19,20^ EGFR mutations have been reported in 10–15% of LC patients in North America and Western Europe and nearly 51.4% of Asians. The L858R point mutation in exon 21 and deletions in exon 19 of EGFR are among commonly reported changes. These mutations accelerate EGFR signaling and trigger malignancy. The main mechanism responsible for resistance to tyrosine kinase inhibitors (TKIs) in EGFR-mutated LC seems to be the incidence of a second mutation, T790M, which is more frequently observed in East Asians. Moreover, V843I, another rare inherited EGFR mutation, has been observed in Asian and non-Asian patients.^21,22^ Finally, ALK gene alterations are found in nearly 5% of NSCLC cases. Many investigations have also demonstrated that alterations in the regulator of G-protein signaling 17 (RGS17) gene on chromosome 6q.55 increase the risk of LC; however, about 20-40% of lung adenocarcinomas show no distinct mutation in this gene.^23,24^

## LC treatment and drug resistance

The type of LC treatment varies according to the subtype of tumor (i.e., SCLC or NSCLC). Conventional methods such as chemotherapy, radiotherapy, and surgical resection are considered as first line therapies. Clinical trials over the past several decades have indicated that the Platinum combined with a third-generation cytotoxic compound such as gemcitabine, vinorelbine, paclitaxel, or docetaxel is the most beneficial and effective chemotherapy regimen for advanced LC.^25^ Using immunotherapy in order to enhance anti-tumor immune response has attracted remarkable attention in recent decades. Immunotherapy by ipilimumab, nivolumab, pembrolizumab, and anti-PD-1 antibody to target the key molecules involved in cancer progression (e.g., programmed death receptor 1 [PD-1] and its ligand [PD-L1] and CTLA-4) has delivered promising therapeutic outcomes in LC patients. To evade the host’s immune system response, tumor cells inhibit T-cells by expressing tumor-specific ligands such as PD-1, PD-L1, and CTLA-4.^26^ In patients with metastatic NSCLC, in whom at least 50% of tumor cells express PD-L1, pembrolizumab has been noted to better increase overall survival in comparison with standard platinum-based chemotherapy.^27^

Chemotherapeutic regimens of LC mostly contain platinum and cisplatin. These agents prevent genome replication by inducing conformational changes and promoting intra-strand cross-links in DNA. Nevertheless, chemotherapy is associated with cytotoxic outcomes such as mitochondrial injuries, cell cycle arrest (at the G2 phase), and reduced ATPase activity and also affects the normal functioning of many healthy dividing cells. As well, it has been established that cancer cells develop resistance against chemotherapeutic agents upon long exposure.^28^

Radiotherapy, as another cancer treatment option, employs high-energy radiation, particularly X-ray, to eliminate cancerous cells. However, healthy cells, especially at the exposure site, also receive the radiation. Hair loss, vomiting, nausea, hematologic abnormalities, and gastrointestinal damage are among common complications of chemo and radiotherapy.^29^

Gene therapy is now acquiring attention in cancer treatment research, particularly because of its lower side effects compared to conventional methods such as chemotherapy. Experimental methods to manipulate the mutated genes involved in cancer progression have shown remarkable success in cancer treatment. Moreover, novel genetic modification tools, unlike traditional approaches of gene therapy, deliver persistent and durable therapeutic effects.^6^

Genome analysis is essential for successful therapeutic intervention in LC. In patients with NSCLC and mutated EGFR, TKIs such as erlotinib and afatinib are effective therapeutics. It has been confirmed that osimertinib, a third-generation EGFR-TKI, is helpful to treat LC patients with T790M mutation.^30^ In patients with ALK translocation (at 2p23), Crizotinib has been suggested as a viable and effective choice.^31^ Nevertheless, most patients ultimately develop resistance to these drugs.

It has been established that the activation of EGFR pathway can accelerate tumor growth and progression in LC. EGFR is expressed in up to 75% of NSCLCs and delivers a poor prognosis.^32^ Two approaches have been suggested to suppress the EGFR pathway: 1) blocking the binding of the ligand to EGFR extracellular domain using a monoclonal antibody (mAb), and 2) suppressing the receptor’s intracellular tyrosine kinase domain. Anti-EGFR mAbs can bind to EGFRs on tumor cells and competitively inhibit the attachment of EGF. Erlotinib (Tarceva), a drug approved by the FDA to be used as a second-line agent for treating LC in 2005, has been reported to prevent tumor progression by blocking EGFR signaling pathway. In 2013, the drug was then designated as the first-line therapy for advanced NSCLCs with mutated EGFR.^33^

**Figure 1:**
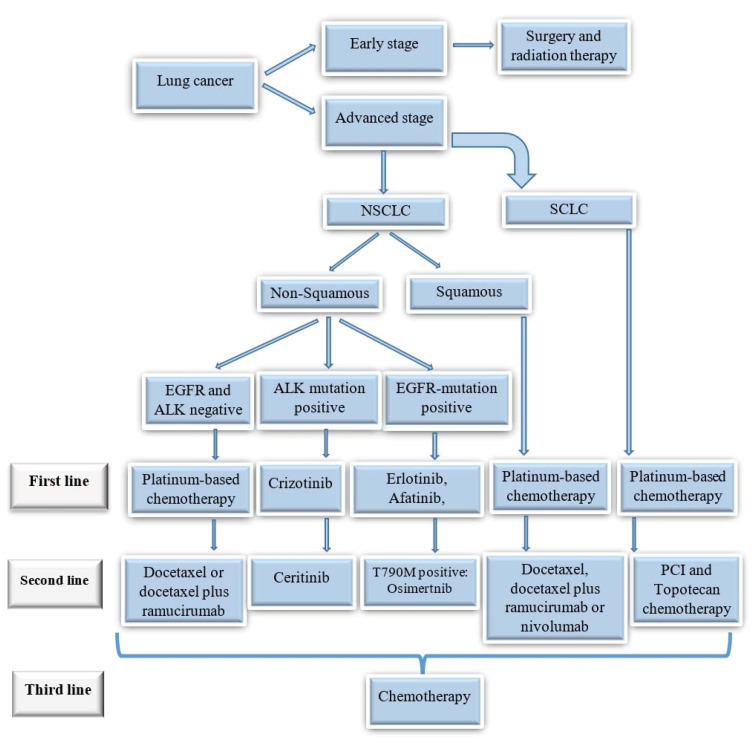
Treatment algorithm for different types of lung cancers.

Although TKIs have significant advantages in treating EGFR-mutated LC, nearly all patients develop resistance to these drugs within two years. A second mutation in exon 20 of EGFR (T790M) was shown to be responsible for resistance to TKIs in LC. The T790M mutation has been detected in ~65% of TKIs-resistant tumors. In this regard, second-generation (e.g., afatinib/Gilotrif, dacomitinib, neratinib) and third-generation (e.g., CO-1686, AZD9291) TKIs have been produced to overcome drug resistance in LC. However, the clinical efficacy of these drugs has not been completely verified yet. New mutations such as C797S have also been identified in the patients treated with third-generation TKIs.^34,35^ Recently, it has been proposed that targeting the MAPK signaling pathway can attenuate the progression of NSCLCs.^36^ Crizotinib, an ALK blocker, was reported to deliver objective response rates up to 75% in ALK-mutated LC (Figure 1).^37^

The role of miRNAs has also been shown in sensitivity or resistance to chemotherapy. The overexpression of miR-106b was noted to suppress polycystin 2 and decrease P-glycoprotein (a main drug expeller molecule) expression. Furthermore, the NSCLC cell lines overexpressing miR-106b have had higher sensitivity to cisplatin. Also, miR-503 was shown to increase cisplatin sensitivity by reducing drug efflux and repressing multiple proteins (e.g. MDR1, MRP1, ERCC1, and Bcl-2) associated with drug resistance. Studies have elucidated a correlation between altered expressions of different miRNAs and sensitivity to gefitinib and erlotinib. For example, miR-21 was described to promote Gefitinib resistance in NSCLC via inducing ERK and ALK pathways and inhibiting PTEN.^38,39^

## CRISPR/CAS system

Decades of research on genome-manipulating approaches have made it possible to precisely modify target DNA sequences at cellular level. This has helped us to understand the roles of different genes in tumorigenesis. The CRISPR-Cas9 gene-editing technology is a novel and high-performance method to effectively treat various genetic disorders, in particular cancers. The CRISPR-Cas system is a part of the prokaryotic adaptive defense system against the invasion of plasmids and viral particles. The Cas is an endonuclease associated with an RNA derived from the CRISPR sequences. This system is activated after exposure to exogenous DNAs from invasive bacteriophages or plasmids and is capable of detecting and eliminating invading molecules. In bacteria and archaea, the Cas protein recognizes foreign invading plasmids or phage DNAs by the help of the sgRNA coming from CRISPR sequences.

CRISPR-Cas systems are categorized into two main classes (i.e., class 1, and 2), and each class is divided into several types and subtypes. Class 1 systems comprise multiprotein effector complexes while class 2 systems contain a single effector protein which combines with the sgRNA and cuts target DNAs. The most common protein applied in class 2 CRISPR-Cas platforms is the Cas9 enzyme which is derived from Streptococcus pyogenes (SpCas9) and employs a small crRNA (spacer) to identify target DNAs and a tracrRNA, as a structural component (scaffold). The tracrRNA within the sgRNA facilitates the interaction of Cas with sgRNA, leading to a correct positioning on target genome sequences. The SpCas9 system recognizes a 20-bp nucleotide sequence which has direct homology with the related sgRNA. In the presence of a 2- to 6-bp protospacer adjacent motif (PAM), the Cas enzyme recognizes DNA sequences via Watson and Crick base pairing between the crRNA and the target DNA. The PAM motif, commonly NGG (“N” being any nucleotide), acts as a location upon which two strands begin to unfold. Different Cas9 enzymes identify different PAM sequences.^40-42^

Exploiting the phage-assisted protein evolution, a SpCas9 was designed to detect general PAM sequences (GAT, GAA and NG) with a higher specificity for DNA.^10^ Recently, an analogue of *Staphylococcus aureus* Cas9 (SaCas9), which has a shorter size compared to the SpCas9, has been identified.^43^ Other Cas9 analogues from *Streptococcus thermophilus* or *Neisseria meningitidis* have also been designed for genome manipulation.^44,45^ In addition to CRISPR–Cas9, CRISPR– Cpf1 is another member of class 2 CRISPRs employed to modify mammalian genome. Cpf1 endonucleases require only one crRNA and one 5ʹ-PAM and exploit a staggered DNA incision sequence.^46^ Class 2 CRISPR systems have three main types: type II, type V, and recently developed type VI. While the recent type employs a driver molecule (i.e., Cas13) targeting single-stranded RNAs (ssRNA), type II and V systems recognize dsDNA.^47,48^

**Figure 2:**
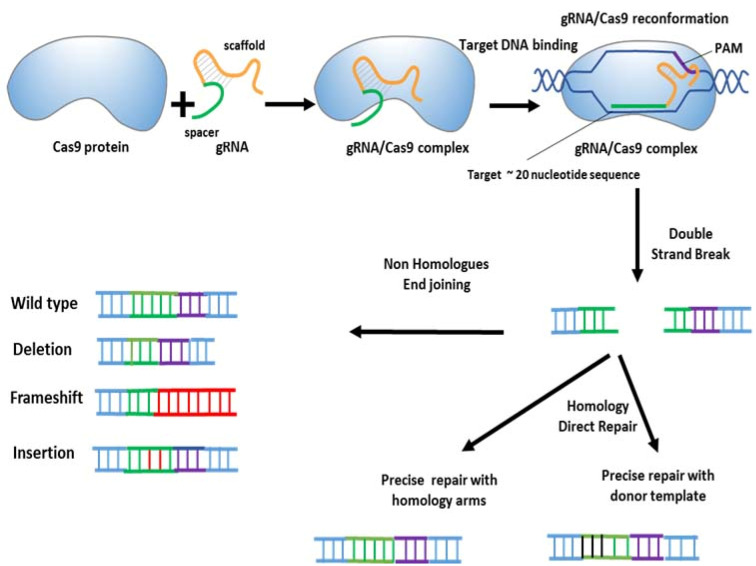
Genome editing by the CRISPR–Cas9 system.

One strategy to improve the efficiency of gene editing is to induce DNA double strand breaks (DSBs) at target genome regions. Generally, DSBs are fixed by DNA repair systems, especially the error-prone non-homologous end joining pathway (NHEJ) pathway which repairs insertions or deletions (indels). DSBs can also be repaired by the homology-directed repair (HDR) pathway which corrects DNA modifications using exogenous single or double stranded donor DNAs or sister chromatids as templates. By employing one sgRNA, the Cas9 disrupts target genes via shifting the reading frame (Figure 2). When two sgRNAs are employed, it is possible to cleave the sequence which is located between two DSBs or induce chromosomal translocations.^11^

Cas9-mediated gene modification requires permanent delivery of CRISPR components. The process can be accomplished by transfecting a plasmid DNA encoding either the Cas9 and sgRNAs or Cas9–sgRNA ribonucleoprotein complexes. Adeno-associated virus (AAV), lentiviruses, or retroviruses can be used to deliver CRISPR components.^49^ The risk of undesirable and harmful mutations is considered as a major obstacle in the clinical application of CRISPR-mediated genome manipulating. The most common cause of miss-targeting is the low specificity of gRNA. So, in designing gRNAs, genetic variabilities among individuals should be taken into consideration.^50^

As mentioned earlier, ZFNs, TALENs, and CRISPR systems are the most common genome engineering technologies that use site-specific endonucleases. CRISPR, ZFNs, and TALENs can induce DNA DSBs which are repaired by NHEJ and HDR pathways. ZFNs and TALENs recognize DNA sequences through protein–DNA interactions. However, the widespread utilization of ZFNs and TALENs has been restricted due to their high costs and the complexity of designing endonucleases. An important advantage of CRISPR compared to other platforms is that the recent technology employs sgRNA-based genome targeting instead of protein-based detection. Using different sgRNAs, the CRISPR-Cas pathway can simultaneously target different genes. A lower number of macromolecules are required to be delivered in the CRISPR-Cas system (i.e., a single Cas9 protein and several sgRNAs) compared to the delivery of multiple ZFNs or TALENs, which increases the complexity and cytotoxicity of the recent strategies. In addition, the CRISPR system has a higher genome-manipulating efficiency in comparison with the other methods.

During genome editing, all types of CRISPR platforms have the risk of off-targeting.^11,51,52^ In this regard, different strategies have been designed to promote the specificity of CRISPR-based editing systems. These include using short sgRNAs, structure-guided designing of Cas9, deactivating Cas9 (dCas9) by FokI enzymes, applying programmable DNA-binding domains such as zinc-finger proteins or TALENs, and introducing chemical alterations to improve specific DNA binding affinity.^10,53,54^ By exploiting several CRISPR activators (CRISPRa) or inhibitors (CRISPRi), the CRISPR-Cas system can also be employed to regulate gene expression.^55,56^

## CRISPR/CAS system in cancer

As mentioned above, the invention of CRISPR–Cas9 systems revolutionized genome manipulating processes and increased their efficiency. The application of this system in cancer gene therapy is due to its ability to induce somatic mutations in cells. In fact, CRISPR-mediated genetic manipulation has led to the discovery of novel therapeutic targets in cancer. Delivering a combination of Cas9 and multiple sgRNAs can simultaneously generate several genetic modifications in tumor cells.^57^ In this regard, the CRISPR method has contributed to the discovery of MELK, a target molecule subsequently leading to the design of OTS167, a MELK blocker, that is currently under study in multiple clinical trials.^58^ In one study, *ex vivo* CRISPR-based disruption of Mll3 in mouse-derived hematopoietic stem and progenitor cells helped to discover the role of Mll3 tumor suppressor in acute myeloid leukemia.^59^ In another study, the plasmids encoding Cas9 and sgRNAs were used to transfect Pten and Trp53 tumor-suppressors to hepatocytes via hydrodynamic gene transmission *in vivo*.^60^ Soda et al applied a CRISPR–Cas9 complex to induce oncogenic chromosomal rearrangements in mice. For this purpose, they delivered an adenovirus harboring Cas9 and two sgRNAs to induce Eml4–Alk (echinoderm microtubule-associated protein-like 4–ALK) inversion. The results confirmed that CRISPR-based somatic genome manipulation can efficiently induce cancer.^61^ The CRISPR system can also be applied to discover mutations responsible for therapy resistance in cancer. For example, NAMPT which encodes nicotinamide phosphoribosyl transferase and is the target of KPT-9274 anticancer agent was identified thanks to the CRISPR technology.^62^ CRISPR-based mutagenesis studies have also resulted in the detection of MEK and BRAF variants conferring resistance to selumetinib and vemurafenib anticancer drugs, respectively.^63^ CRISPR-based rearrangement of CTCF binding sites, which are often mutated in multiple types of cancers, was shown to change the activities of their promoters and enhancers, as well as the gene topology.^64^ The CRISPR-Cas9 method has been used to deactivate PD-1 and endogenous TCR to prevent the immune evasion of tumor cells. In one study, the gene encoding PD-1 protein was switched oﬀ by CRISPR-Cas9, which enhanced the anti-tumor activity of lymphocytes.^65^ The beneficial effects of the CRISPR/Cas system in studying different aspects of LC such as tumor cell modeling, treatment, diagnosis, and drug resistance have been discussed below.

## CRISPR-CAS9 system in LC modelling

LC modelling and engineering tumor cells are among the most important applications of CRISPR-based techniques. CRISPR-mediated knockout of tumor suppressor genes, specially through the NHEJ process, has provided the opportunity to fabricate natural tumor cells.^66^ Guernet et al utilized a CRISPR-barcoding strategy to predict different mechanisms of resistance to EGFR inhibitors in NSCLC. First, the CRISPR-Cas9 system was exploited to insert several point mutations into a distinct genomic region in order to label and trace tumor cells. To create a new model of EGFR-inhibitor resistant NSCLC, they generated a specific sgRNA and a donor single-stranded DNA oligonucleotide (ssODN) harboring the T790M mutation. A few additional silent mutations were also inserted to provide a genetic barcode. To prevent the two DNA sequences to be inserted into wrong locations, EGFR inhibitor-sensitive PC9 cells were transfected with a CRISPR/Cas9 plasmid encoding Cas9 and one sgRNA, together with one of the two ssODNs. It was revealed that treatment with gefitinib increased the ratio of EGFR-T790M to EGFR-T790T, indicating an increase in resistance to the EGFR inhibitor.^67^

Sánchez-Rivera et al applied the CRISPR/Cas approach to investigate the function of a number candidate genes in autochthonous mouse models of LC. To rapidly insert oncogenes in the lung, they used pSECC, a lentiviral-based system that integrates both the CRISPR system and Cre recombinase. Lung tumors were induced by transfecting Kras^LSL-G12D/1^ and Kras ^LSL-G12D/1^+ p53^fl/fl^ mice with pSECC vectors harboring sgRNAs for phosphatase and tensin homologue (Pten), a reverse regulator of oncogenic PI(3)K/Akt pathway, and NK2 homeobox 1 (Nkx2-1), a key regulator for lung differentiation and development. Ten weeks after the transfection, all the animals that expressed sgRNAs targeting Pten or Nkx2-1 developed tumors with significant histopathological diversity compared to controls. The animals transfected with the sgNkx2-1-pSECC also developed mucinous adenocarcinomas characterized with the presence of elongated cells, high mucin production, and glandular rearrangements. The animals infected with the sgPten-pSECC showed decreased expression of Pten protein in 74% of tumors, which was associated with elevated pAkt (S473), a downstream marker indicating the activation PI(3)-kinase pathway. Collectively, these data indicated that CRISPR/Cas9-mediated gene editing was effective in inducing loss-of-function mutations in this model. The researchers; however, observed a negligible rate of off-target editing in this system.^68^

Chromosomal rearrangements play a key role in the pathogenesis of human cancers. A recently discovered example is the ALK -EML4 fused gene resulting from an inversion on the short arm of chromosome 2 [inv (2) (p21p23)]. The EML4–ALK fusion oncogene is commonly expressed in NSCLCs and is clinically important because it confers sensitivity to ALK blockers. In one study, Maddalo et al applied viral-mediated insertion of the CRISPR/Cas9 system into somatic cells to create the EML4–ALK fusion gene and trigger LC. The expression of the fused oncogene resulted in the typical histopathological and molecular features of ALK+ human NSCLCs which responded to treatment with ALK inhibitors.^69^

## CRISPR-CAS9 in LC treatment

Many studies have investigated the efficacy of the CRISPR system for treating LC. Lu et al performed a phase I clinical trial to evaluate the safety of CRISPR/Cas9-based knockout of PD-1 gene in autologous T lymphocytes in patients with metastatic NSCLC. In this clinical trial, the PD-1 deficient T cells manipulated by the CRISPR/Cas9 system showed an excellent safety profile. However, further studies are necessary to evaluate the effective doses and related immune responses.^70^

Interleukin-16 (IL-16), a proinflammatory cytokine produced by blood mononuclear cells and CD4+ T cells, was originally identified as a lymphocyte chemoattractant. IL-16 is also expressed in airways and by alveolar epithelial cells. This cytokine participates in the cellular activation and migration mediated by CD4 membrane protein. Many types of cells including epithelial, dendritic, and mast cells express CD4; therefore, they can respond to this potent chemoattractant.^71^ In one study, Blake et al ablated CD 9 surface protein on human LC cells using the CRISPR/Cas system and evaluated the chemoattractant activity of IL-16. They found that while wild-type A549 cells migrated in response to IL-16, CRISPR/Cas-mediated CD 9-depleted cells showed remarkable reduction in migration. Therefore, blocking CD 9 expression by the CRISPR-Cas system can be considered as an effective therapeutic strategy in LC.^72^

The T790M mutation in the EGFR gene suppresses response to first-line TKIs in LC. Another mutation, L858R, has been identified in 42.5% of EGFR-mutated lung adenocarcinomas among Asians patients. In the recent mutation, T>G substitution occurs in exon 21 of EGFR, inducing the persistent activation of its kinase domain activating pro-survival signal transduction pathways.^73^ Cheung et al showed that the CRISPR/Cas9-mediated targeting of L858R mutation in LC cells triggered the selective removal of L858R mutant cells and decreased cancer progression.^74^ Tang et al employed the CRISPR/Cas9 system to either correct or destroy EGFR-mutated cells in biopsy samples from patients with NSCLC. They used a virus-delivered CRISPR/Cas system and sgRNAs to target distinct sequences in mutated exons (i.e., L858R in exon 21, E19del in exon 19, or T790M in exon 20). Donor DNAs containing the wild-type sequence of exon 19 or 21 and their right and left homologous arms were substituted with mutated sequences or exons via homologous recombination (i.e., HDR). This method was highly efficient for the studied EGFR mutations (E19del and L858R). The DNA constructs of the CRISPR/Cas9 system were incorporated into a virus and delivered to patients via either the trachea (for localized cancers) or intravenous injection (for metastatic cancers).^75^

Continuous *de novo* fatty acid synthesis regulated by the acetyl-CoA carboxylase is necessary for tumor growth. Svensson et al demonstrated that the CRISPR–Cas9-mediated deletion of ACC1 in NSCLC completely inhibited fatty acid synthesis and significantly decreased the viability, proliferation, and tumorigenesis of cancerous cells. In fact, endogenous ACC2 did not compensate for the loss of ACC1 in these cells, indicating an essential role for ACC in the metabolic activity of NSCLC and its therapeutic potential in this cancer.^76^

Suppressing the p53 tumor suppressor by Mdm2 (E3 ubiquitin ligase) plays a key role in cancer pathogenesis. Therefore, compounds that prohibit p53-Mdm2 interactions (such as nutlin and RITA) may be beneficial for treating cancers. Nutlin blocks the p53-binding pocket of Mdm2, and RITA binds to the N terminus of p53, which interacts with Mdm2.^77^ To identify whether or not the actions of Nutlin and RITA are dependent on different alleles of *TP53*, Wanzel et al targeted this gene in LC cells using a CRISPR-Cas9–based approach and specific sgRNAs which were cloned into pX330-U6-Chimeric_BB-CBh-hSpCas9 and lentiCRISPR v1 vectors. They showed that Nutlin blocked tumor growth while p53 was dispensable for RITA action.^78^

Protocadherin 7 (PCDH7), a member of the Cadherin superfamily, is a transmembrane receptor which is commonly overexpressed in lung adenocarcinomas and predicts a poor prognosis.^79^ Zhou et al applied a CRISPR/Cas9 somatic genome editing system in Kras^LSL-G12D^; Tp53^fl/fl^ (KP) mice to evaluate the consequences of PCDH7 deletion. The inhibition of PCDH7 in KP mice remarkably decreased lung tumor progression and reduced phospho-activation of ERK1/2. These results confirmed a pivotal oncogenic action for PCDH7 and highlighted its potential to be subjected to gene editing in LC.^80^

Candi et al assessed the applicability of a CRISPR interference system to suppress ΔNp63 in lung and esophageal SCCs. *TP63* gene executes a role similar to that of *TP53* tumor suppressor. The former gene encodes ΔNp63 and is known as a potent oncogene in SCCs.^81^ To develop an all-in-one adenoviral vector, the researchers generated a CRISPRi system harboring a tandemized gRNA expressing inactivated Cas 9 to target and suppress the expression of ΔNp63 in SCC cells. It was shown that the Ad-CRISPRiΔNp63 significantly suppressed cellular proliferation and induced apoptosis in lung and esophageal squamous carcinoma cells *in vitro*. Moreover, the recent vector markedly prevented tumor growth in a xenograft mouse model of lung SCC. These findings suggested that ΔNp63 suppression by the CRISPRiΔNp63 vector could be considered as a potential strategy for treating lung and esophageal SCCs.^82^

## CRISPR-CAS9 and LC drug resistance

Upon prolonged exposure, lung adenocarcinoma cells usually promote resistance against TKIs such as erlotinib and gefitinib. Cancer cell lines with distinct mutations can help to identify the mechanisms of drug resistance. On the other hand, the CRISPR/Cas9 programmable nuclease complex can be applied to introduce specific mutations into different cancer cell lines. In half of cases, the EGFR T790M mutation is associated with resistance to TKIs in LC.^83^ In an experimental study, Park et al used the CRISPR/Cas9 system to introduce the T790M mutation as well as an exon 19 deletion into the EGFR gene in PC9 human LC cell line. In the HDR process, the researchers used an oligonucleotide containing two 50-bp outer arms homologous to the ﬂanking regions of the endogenous EGFR. EGFR pyrosequencing and peptide-nucleic acid clamping proved that the PC9 cells carrying the designed CRISPR/Cas 9 system had higher rates of the T790M mutation compared to the cells long-exposed to gefitinib (PC9-G). Accordingly, the CRISPR/Cas 9-manipulated cells showed a higher resistance to gefitinib than PC9-G cells and were highly susceptible to AZD9291, a 3rd-generation EGFR TKI.^84^

NSCLC resistance to radiotherapy is a major cause of treatment failure. Caveolin-1 (Cav1), a transmembrane protein, has been shown to be markedly overexpressed in several radioresistant tumors. Samanta et al manipulated CAV1 expression in NSCLC cells and investigated the resistance of these cells to radiotherapy. They showed that the CRISPR/Cas9-mediated knockout of Cav1 increased the sensitivity of NSCLC cells to radiation. Also, transfecting radiosensitive A549 cells with Cav1 expressing lentivirus conferred dose-dependent radio-resistance to these cancer cells. These findings clarified that Cav-1 could be considered as a new prognostic biomarker to predict resistance to radiotherapy in NSCLC.^85^ Recent studies have indicated that paraoxonase 2 (PON2), a lactonase/arylesterase with antioxidant properties, is significantly overexpressed in cancer tissues of NSCLC patients. PON2 overexpression has been shown to contribute to the resistance of NSCLC cells to chemotherapeutic agents and CRISPR/Cas-mediated PON2 downregulation to suppress the proliferation of these cells.^86^

## CRISPR-CAS9 in LC pathogenesis

The discovery of CRISPR technology facilitated the detection of tumorigenesis mechanisms of LC. Eichner et alinvestigated the role of AMPK in murine Kras^G12D^-induced NSCLC. AMPK is considered as an indicator of low cellular energy and can either inhibit or augment tumor growth depending on the type of the tissue. NSCLC tumors usually harbor several inactivating mutations in the LKB1 tumor suppressor which is the predominant upstream stimulator of AMPK and 12 related kinases. It was revealed that unlike the deletion of LKB1, the removal of AMPK by CRISPR/Cas9 did not promote tumor growth in Kras^G12D^ LC. Furthermore, the deletion of AMPK in Kras^G12^D p53^f/f^ tumors decreased lung tumor growth. AMPK is able to induce Tfe3 dephosphorylation and subsequently upregulate lysosomal gene expression, which is required for NSCLC progression.^87^

The friend leukemia virus integration 1 (FLI1) is highly expressed in SCLC and also in hematopoietic cells, endothelial cells, and fibroblasts. FLI1 is considered as a major driver of hematological malignancies.^88^ Li et al used a CRISPR/Cas9 gene editing system to deactivate the FLI1 gene, applying two sgRNAs targeting the exons 2 and 3 of this gene. The knockdown of FLI1 via the CRISPR/Cas9 approach more efficiently prevented the cellular proliferation and migration of SCLC cells compared to shRNA-dependent knockdown that targeted the 3’ region of FLI1 mRNA.^89^

In another study, Wu et al used the CRISPR/Cas9 system to introduce inactivating somatic mutations into a number of tumor suppressors in a Kras^G12D/+^ mouse model. They showed that the functional removal of five genes including Utx, Ptip, Acp5, Acacb, and Clu remarkably accelerated lung tumorigenesis. These genes are usually down-regulated in human LC and have an impact on the survival of LC patients.^90^

Platt et al utilized an AAV vector to deliver a CRISPR-Cas9 system to the lungs of mice. They showed that loss-of-function mutations in p53 and LKB1 and HDR-mediated Kras^G12D^ mutations (KPL) resulted in macroscopic adenocarcinoma tumors. AAV9-KPL-transfected mice developed multiple grade I and II bronchial alveolar adenomas one month after the injection. These tumors progressed to grade III and in some cases to grade IV adenocarcinomas after two months. Many of these tumor cells were positive for Ki67, an indicator of active cell cycle.^66^

The most important tumor suppressors associated with the progression of LC are p53 and LKB1 kinase. Marignani used a Cre-dependent Cas9 to generate mice models bearing KRas^G12D^ and knocked out Lkb1 and p53 and showed that the simultaneous removal of these tumor suppressors activated mTOR, a marker of tumorigenesis and a pro-survival pathway, and increased the acetylation and methylation of histones 3 (H3) and H4, indicating a poor prognosis.^91^

The tumor suppressor activity of the Gene 33 (Mig6, ERRFI1) has been confirmed in LC. This tumor suppressor suppresses the tyrosine kinases associated with the EGF receptor. Park et alhave recently reported that the absence of this protein promotes the neoplastic transformation of the lung epithelial cells exposed to hexavalent chromium [Cr(1)], a human lung carcinogen. They utilized single-cell RNA sequencing to determine the expression of Gene 33 in the BEAS-2B lung epithelial cells exposed to Cr (1), with or without CRISPR/cas9-based knocking out of Gene 33. Gene 33 deletion significantly decreased the number of cells in the G2/M phase of the cell cycle and increased cell migration.^92^

Ng et al transfected a Cre-activated allele of Cas9 into Trp^53flox/flox^, Rb1^flox/flox^ murine models of SCLC using the adenoviral vectors harboring the Cre recombinase and sgRNAs targeting either p130 (Rbl2) or p107 (Rbl1). The recent genes (Rb11 and Rb12) are commonly mutated in a subset of human SCLCs. The loss of p107 or p130 expression in these tumors was proved via immunohistochemical staining. The CRISPR-mediated loss of p130 and p107 in Trp53^flox/flox^ and Rb1^flox/flox^ animals accelerated tumor progression. Therefore, in this study, CRISPR-Cas9 approach verified p130 and p107 as tumor suppressors in SCLC.^93^

## Conclusion and perspectives

LC is the most common cause of cancer-related death worldwide and is usually diagnosed at advanced phases. The five-year survival rate of LC is only 17%. Surgery, chemotherapy, and radiotherapy are major therapeutic modalities of LC, but these methods are generally associated with unwanted cytotoxic outcomes towards healthy cells and tissues (e.g., mitochondrial dysfunction, cell cycle arrest, ATPase suppression, hair loss, vomiting, nausea, hematological abnormalities, and gastrointestinal problems). Furthermore, LC tumor cells develop resistance to chemotherapeutic agents upon long exposure. Gene therapy, which particularly has fewer side effects, is considered as an effective alternative for conventional cancer treatments such as chemotherapy. Genome manipulation, unlike the traditional approaches of gene therapy that have temporary effects, has shown promising and long-lasting outcomes in cancer treatment. Genome editing systems such as the CRISPR-Cas9 can be used to introduce various genetic modifications (e.g., missense point mutations, chromosomal rearrangements, epigenetic changes) into different types of cells. In this regard, the CRISPR-Cas9 system has been the subject of tremendous efforts to investigate the roles of pathogenic genes in various cancers. In LC, the CRISPR-Cas9 system has widely been applied to suppress EGFR expression, bypass TKIs resistance, repress ALK-activated cells at early stages, inhibit K-RAS oncogene, and finally induce desirable epigenetic alterations. The CRISPR gene manipulation system has shown advantages such as low cost, feasibility, and high efficiency compared to traditional gene therapy methods such as ZFN and TALENs. So, along with conventional surgery, radiation therapy, and chemotherapy, this technology provides a great opportunity to improve therapeutic outcomes in LC.

## Ethical Issues

Not applicable.

## Conflict of Interest

Authors declare no competing interests.
